# Towards Forensic DNA Phenotyping for Predicting Visible Traits in Dogs

**DOI:** 10.3390/genes12060908

**Published:** 2021-06-11

**Authors:** Cordula Berger, Josephin Heinrich, Burkhard Berger, Werner Hecht, Walther Parson, on behalf of CaDNAP

**Affiliations:** 1 Institute of Legal Medicine, Medical University of Innsbruck, 6020 Innsbruck, Austria; josephin.heinrich@live.com (J.H.); burkhard.berger@i-med.ac.at (B.B.); walther.parson@i-med.ac.at (W.P.); 2 Institute of Veterinary Pathology, Justus-Liebig-University Giessen, 35390 Giessen, Germany; wernerhecht@web.de; 3 Forensic Science Program, The Pennsylvania State University, University Park, PA 16801, USA

**Keywords:** domestic dog (*Canis familiaris*), canine DNA phenotyping, forensic, proof of concept study

## Abstract

The popularity of dogs as human companions explains why these pets regularly come into focus in forensic cases such as bite attacks or accidents. Canine evidence, e.g., dog hairs, can also act as a link between the victim and suspect in a crime case due to the close contact between dogs and their owners. In line with human DNA identification, dog individualization from crime scene evidence is mainly based on the analysis of short tandem repeat (STR) markers. However, when the DNA profile does not match a reference, additional information regarding the appearance of the dog may provide substantial intelligence value. Key features of the dog’s appearance, such as the body size and coat colour are well-recognizable and easy to describe even to non-dog experts, including most investigating officers and eyewitnesses. Therefore, it is reasonable to complement eyewitnesses’ testimonies with externally visible traits predicted from associated canine DNA samples. Here, the feasibility and suitability of canine DNA phenotyping is explored from scratch in the form of a proof of concept study. To predict the overall appearance of an unknown dog from its DNA as accurately as possible, the following six traits were chosen: (1) coat colour, (2) coat pattern, (3) coat structure, (4) body size, (5) ear shape, and (6) tail length. A total of 21 genetic markers known for high predicting values for these traits were selected from previously published datasets, comprising 15 SNPs and six INDELS. Three of them belonged to SINE insertions. The experiments were designed in three phases. In the first two stages, the performance of the markers was tested on DNA samples from dogs with well-documented physical characteristics from different breeds. The final blind test, including dogs with initially withheld appearance information, showed that the majority of the selected markers allowed to develop composite sketches, providing a realistic impression of the tested dogs. We regard this study as the first attempt to evaluate the possibilities and limitations of forensic canine DNA phenotyping.

## 1. Introduction

DNA phenotyping in a forensic context was developed as meaningful enhancement to standard human DNA profiling, where STRs are mainly used to identify individuals [1,2]. In cases where DNA profiles do not match a suspect’s profile or a criminal DNA database record, forensic DNA phenotyping (FDP) aims to predict externally visible characteristics of a person by analyzing suitable DNA markers. It provides new investigative leads irrespective of the presence of any other information such as eyewitness testimonies [1]. DNA-based appearance prediction within forensics started in the early 2000s [3] and so far, tests on human iris, hair, and skin pigmentation were successfully validated for routine casework investigations [1,4,5,6,7,8]. Their implementation within legislative frameworks of different countries is now discussed intensively [9].

In this study, we aim at outlining an approach for Canine DNA phenotyping in forensic settings as it is an appealing idea to test if the concept of FDP can be transferred to non-human DNA. As a first step, we generated proof of concept data to explore ways and possibilities for developing a molecular genetic tool to predict externally visible traits of dogs from DNA customized to the special needs of forensic issues. The choice fell on the domestic dog because of its extreme morphological diversity and its forensic significance.

The diverse appearance of dogs is the result of a long-standing and intensive domestication process. Although still being discussed with controversial arguments, it is broadly accepted that all contemporary dogs were domesticated from the Eurasian grey wolf (*Canis lupus*) and that they accompanied humans over millennia [10,11,12,13,14,15,16,17,18,19,20,21]. Dogs have performed a variety of roles starting from ancestral dogs that were primarily valued for their hunting or protective skills [22,23]. The desire to develop dogs with particular physical traits was the driving force behind selective breeding practices. During the last 200 years, the focus of breeding changed from working ability to outward appearance and resulted in the formation of modern dog breeds [24,25,26]. At present, more than 400 breeds exist, and this diversity is even leveraged by inter-breeds and mongrels. Dogs exhibit a vast array of phenotypic traits, with varying body sizes, skull shapes, hair structure, coat colours, etc. [27,28,29,30,31,32,33,34,35,36,37,38,39,40,41,42,43,44,45,46,47,48,49,50,51,52,53,54,55,56]. As a result, even an untrained eye can easily identify and describe some significant characteristics of a dog. This leads one to expect that the output of a canine DNA phenotyping test could provide practical information about the appearance of a dog of interest, which is comparable to an eye-witness testimony.

Today, there is an estimated number of almost 700 million to 1 billion dogs worldwide [57,58]. The dog density varies considerably among countries, with much higher numbers across the United States or Europe [57]. In these countries, dogs are kept almost exclusively within the home, underlining the dog’s major role as a companion. This explains why canine-source material is relevant to diverse forensic cases. Dogs can cause accidents or attack humans, wildlife and domestic animals. For a recent overview concerning dog-related fatalities, see [59]. Probably forensically more relevant, and more challenging to analyse, are cases in which the transfer of canine DNA evidence occurs. Such instances can identify links between victims, suspects, and/or crime scenes and thus contribute to generating evidence in human forensic cases. Due to the close contact with humans, canine transfer samples can be plentiful. This may include, for example, saliva on trousers or hair left on the back seat of a vehicle. As dogs shed more hairs than humans, the probability of finding dog hairs can be higher than finding hairs of the dog owner.

DNA fingerprinting methods provide strong evidence for individual identification of the involved dog(s). Accordingly, a number of approaches have been published using canine STR loci for dog individualization from crime scene evidence [60,61,62,63,64,65,66,67,68] and guidelines have been established for forensic validation requirement [69,70]. STR profiling allows to answer questions such as: “was this dog the perpetrator of the attack?” or “does the hair found on the victim originate from the suspect’s dog?”. However, cases where crime scene DNA does not match a suspected dog or where reference material is not available occur frequently. Consequently, statements such as “the attack was perpetrated by a large dog with black coat colour” or “the hair comes from a black/white spotted dog with long fur” would be typical examples for helpful leads. Traditionally, such statements come from eyewitness reports. However, eyewitness testimony is not always available and can be subjective or unreliable [71,72,73,74]. DNA analysis, particularly when tailored toward canine DNA phenotyping, could counteract these limitations by answering questions similar to the above-mentioned examples based on an objective and quantifiable methodology.

## 2. Materials and Methods

### 2.1. Sampling, Documentation and DNA Extraction

Our sample collection was established based on direct inquiries to private dog owners, by visiting dog shows, dog schools and dog breeders. Buccal swabs were taken by the dog owner to minimize disturbing effects to the animals. Alphanumeric unique sample IDs were used for unambiguous sample assignment. Metadata, including all relevant externally visible characteristics, were recorded as displayed exemplarily in Table S1. In addition, tissues from dead dogs were collected at the Institute for Veterinary Pathology, Justus-Liebig-University Gießen, Germany, as described in [61]. In total, 84 dog samples were included in the study.

DNA was extracted from buccal swabs according to routine procedures on a Qiagen EZ1 Advanced XL Nucleic Acid Automated Purification System (Qiagen, Hilden, Germany) using the DNeasy Blood & Tissue Kit (Qiagen) or using Gentra Puregene reagents and protocols (Qiagen), according to the manufacturers’ recommendations. The DNA of the tissue samples was extracted in a class 2 biological safety cabinet using the Gentra Puregene Tissue Kit (Qiagen, Hilden, Germany) [61,75]. Nuclear DNA quantities were determined with a spectral photometer (Nanodrop 2000; Peqlab GmbH, Erlangen, Germany) or by applying a quantitative real-time PCR assay according to [76]. The assay was performed in a total volume of 10 μL on an AB 7500 Fast Real-Time PCR System (Thermo Fisher Scientific [TFS], Waltham, MA, USA).

### 2.2. Marker Selection and Study Design

A total of 21 markers were selected, comprising 15 SNPs and six INDELS. The latter two were microINDELS (each with a length of 3 bp), one was of intermediate-size (167 bp) and three belong to SINE insertions. The complete marker set is listed in Table 1, which contains relevant molecular genetic information as well as metadata regarding the related phenotypes. The primer sequences and positions according to CanFam3.1 are provided in Table S2a.

The implementation of the proof of concept study was divided into three phases or experimental tests that built on one another: the pilot test, the marker test, and the blind test. Within each test, Sanger sequencing of all markers was carried out on a set of canine DNA samples in order to create molecular data for downstream phenotype interpretations.

### 2.3. PCR Amplification and Sanger Sequencing

Where possible, primer sequences were directly adopted from published data or were redesigned to reduce amplicon lengths. Detailed information on the primers is provided in Table S2a. Primer design was performed with the OligoAnalyzer Tool (Integrated DNA Technologies, Coralville, IA, USA) based on the CanFam3.1 (EMBL-EBI, Hinxton, Cambridgeshire, UK) reference sequences and was checked for secondary structure formation using Mfold (University at Albany, NY, USA).

PCR amplifications were carried out in 20 µL assays, containing 0.4 µL 50X Advantage 2 Polymerase Mix, 2 µL 10X Advantage 2 PCR Buffer (both Takara, Kyoto, Japan), 0.25 mg/mL BSA (Serva, Heidelberg, Germany), 200 µM each dNTP (TFS), 0.6 µL of each 10 µM primer (Microsynth, Balgach, Switzerland, Table S2a). Two to 30 ng of DNA extract were used for amplification. Thermal cycling was performed on a DNA Engine Dyad thermal cycler (Bio-Rad, Hercules, CA, USA) or a Gene Amp PCR System 9700 (TFS), comprising initial denaturation at 95 °C for 2 min, followed by 37 cycles of 95 °C for 15 s, 58 to 67 °C for 30 s (see Table S2a) and 72 °C for 45 s. PCR products were purified using ExoSAP-IT (Amersham, Bucks, UK) according to the manufacturer’s recommendations.

Sanger sequencing was accomplished by combining 2 µL of BigDye Terminator v1.1 Ready Reaction Mix, 2 µL 5X Sequencing Buffer (both TFS), 1 µL 5 µM amplification primer and 5 µL amplification product to a total volume of 10 µL. Cycling conditions were 95 °C for 2 min, and 30 cycles of 96 °C for 1 min, 50 °C for 5 s and 60 °C for 4 min. Products were purified using the Performa DTR Ultra 96-Well Plate Kit (EdgeBio, San Jose, CA, USA) according to the manufacturer’s recommendations. Capillary electrophoresis was performed on an AB 3500xl Genetic Analyzer using POP6, 50 cm capillary arrays and default instrument settings (all TFS). Sequencing data were analysed using Sequencing Analysis Version 5.4 (TFS) and Sequencher Version 5.1 (GeneCodes, Ann Arbor, MI, USA). SNP data were formatted in Excel 2016 (Microsoft Corporation, Redmond, WA, USA).

The genotypes relevant for predicting visible traits comprised single nucleotide polymorphisms (SNPs) and insertion/deletion markers (INDELS) including short interspersed nuclear elements (SINEs; sequences are provided in Table S2b). The polymorphic sites were addressed by sequence analysis of the amplified DNA fragments. The interpretation of the allelic states at homo- and heterozygous SNP sites proved unambiguous. The same held true for short INDELS (up to three base pairs). However, long INDEL markers, ASIP SINE, MITF SINE, PMEL SINE, and the RSPO2 insertion caused difficult-to-interpret raw data when heterozygous. In these cases, short and long amplicon sequences (derived from both, the allele without insertion and with insertion) overlapped and caused out of phase sequence data. For interpretation of the heterozygous state, the overlapping nucleotides within the chromatogram—reflecting both sequence variants—were considered separately (see Figure S1a–d).

## 3. Results

**Figure 1:**
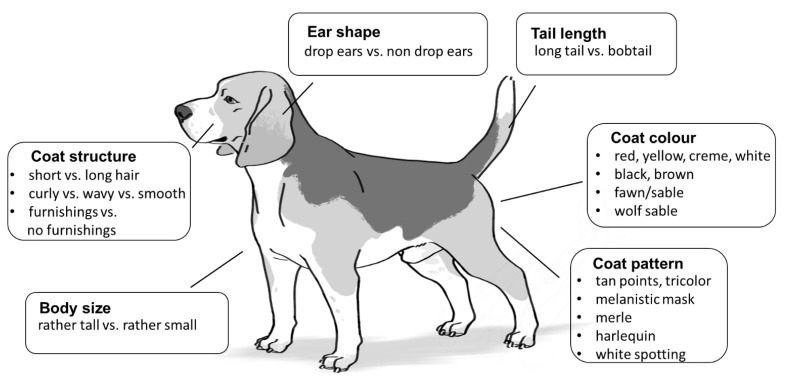
Schematic illustration of the trait categories selected to deduce a dog’s appearance by applying canine DNA phenotyping. The six trait categories include the most obvious characteristics of a dog that can easily be recognized and described even by an untrained eyewitness. Each box refers to one trait category and contains clearly distinguishable externally visible characteristics predictable from a particular genotype. The listings should provide an overview of the standardized terminology used here to characterize the appearance. vs.—versus (picture copyright: Larissa Hasenheit).

For a phenotypic description of individual dogs, genetic markers for the following six traits were selected: (1) coat colour, (2) coat pattern, (3) coat structure, (4) body size, (5) ear shape, and (6) tail length. In total, a set of 21 markers was applied (Table 1). Figure 1 provides a schematic representation of the possible phenotypic manifestations that could, in principle, be deduced from this marker set. This information is based on the published explanatory power of the markers (Table 1). In general, our results confirmed the published effects of alleles at the tested loci on the phenotypic appearance of dogs.

### 3.1. Pilot Test

The pilot test included DNA samples from twelve dogs selected from a sample collection comprising approximately 1200 dogs [75]. Each of the 12 samples were analysed in 21 markers, resulting in 252 genotypes. This test was performed to verify the SNP and INDEL positions provided in the literature and to optimize the laboratory workflow, including PCR and sequencing conditions as well as primer redesign, if necessary (see Table S2a). All 21 markers were successfully sequenced and all SNPs/INDELS could be unambiguously assigned. In all but three markers (PMEL, PSMB7, and T-box), all previously described allelic states were found within the pilot test sample set (Table S3a).

### 3.2. Marker Test

In the marker test, the reliability of predicting the phenotype for each of the 21 markers representing the six trait categories was determined. Therefore, the test samples were selected from the full sample collection (*n* = 1200) with respect to the phenotypical appearance of the dogs to include the different phenotypic manifestations of a particular trait. A detailed list of possible genotypes at all markers and the resulting genetic-based phenotype prediction is given in Tables S4a–k.

The following presentation of the marker test outcomes is accompanied by an overview of the genetic basis and nomenclature commonly used for each trait. The simplified chart given in Figure S2 summarizes the dominance hierarchy of loci and alleles involved in the manifestation of coat colour and pattern.

#### 3.2.1. Coat Colour and Coat Pattern

The nomenclature for the genetics of coat colours and coat patterns in dogs is based on the system established by [77] and with later adaptations (e.g., [38,55,78,79]). The term “Locus” is preceded by an alphabetical character which refers to the main characteristic realized by the particular locus (e.g., A-Locus: “A” stands for “Agouti”). Since the introduction of this naming convention by Little (1957) [77], all loci were assigned to genes, as described below.

As in most mammals, the synthesis of eumelanin (black, brown) or phaeomelanin (yellow, red) is regulated by the two genes *MC1R* (E-Locus) and *ASIP* (A-Locus). In addition to these two genes, *CBD103* (K-Locus) is of particular importance for black fur [28,80,81]. The genes *MITF* (S-Locus), *MLPH* (D-Locus), *PMEL* (M-Locus), and *PSMB7* (H-Locus) modify the coat colour and pattern that is provided by the three main loci described above and are therefore called “modifiers” [82].

For testing the coat colour markers, one set of sixteen dog samples was used. It consisted of five solid brown, two solid black, two fawn/sable with white spotting, and two brown dogs with white spotting, as well as one wild type agouti, one brown sable, one black with white spotting, one black merle, and one blue (grey) dog.

**E-Locus** (“Extension”); see Table S4a: The *MC1R* (Melanocortin 1 receptor) gene is epistatic to the K- and A-loci and responsible for a red, yellow, creme, and sometimes white coat colour in dogs (genotype: e/e) [31,32,33,34,83]. Alleles of the E-Locus are the dominant E (establishing a brown or black coat colour), and the recessive e. The allele for the coat pattern “melanistic mask” E^m^ is dominant over the E allele. Therefore, a melanistic mask is present in all E^m^ dogs, even if phenotypically not so well recognizable as in solid black dogs [79].

**K-Locus** (“dominant black”); see Table S4b: The *CBD103* (β-defensin) gene is responsible for black coat colour [28]. The effect of the K-Locus is dependent on the E-Locus and is only expressed when at least one E allele is present in a genotype. The dominant K^B^ allele inhibits the expression of the A-Locus. That results in solid coloured phenotype when no modifier gene (e.g., *MLPH*) is expressed [40,79]. The coat pattern “brindle” (K^br^), in which stripes of red-yellow hair alternate with black-brown hair, was not included in this study.

**A-Locus** (“Agouti”); see (Table S4c): The Agouti signaling protein gene has four alleles identified so far, hierarchically ordered according to their dominance: A^y^ (fawn/sable) > a^w^ (agouti/wild type- wild type, black banded hairs) > a^t^ (tan points) >a (recessive black) [27,29]. The A-Locus is expressed when the K-Locus is homozygous for the recessive k^y^ allele. The coat pattern “sable” arises from hairs with tips darker in colour than the light-coloured base. Three main sable patterns are common in dog breeds: (1) clear sable—completely fawn dogs with just a few eumelanin (black/brown) hairs; (2) tipped sable—fawn dogs with eumelanin (black/brown) hairs usually on the back, head, ears and tail; (3) shaded sable—fawn dogs with eumelanin (black/brown) hairs covering the top of the head, ears and back; shading can be very light or very dark and distinct [http://www.doggenetics.co.uk/tan.html#sable] (accessed on 10 June 2021).

Agouti (a^w^—“w” stands for “wild type”) is characterized by hairs showing alternating colours along the hair shaft (“banding”; Fawn/black or brown).

**B-Locus** (“Brown”); see Table S4d: The gene *TYRP1* (Tyrosinase-related protein 1) affects only eumelanin, causing all black areas to turn to a brownish colour [33,55]. Brown is expressed by recessive alleles. It can be expressed when two of the three brown alleles are present in a genotype (b^s^/b^s^, b^s/^b^d^, b^s^/b^c^, b^d^/b^d^, b^d^/b^c^, b^c^/b^c^) [55]. The gene also affects the colour of the nose and eyes, making them brownish (liver).

**D-Locus** (“Dilution”); see Table S4e: The *MLPH* (Melanophilin) gene affects both eumelanin and phaeomelanin [30,56,84]. As the allele causing dilution is recessive, a dilution is only expressed when a dog is homozygous for this allele. In this case, a black dog will become dark grey (also called “blue”), and a brown dog will become pale cream-brown (“Isabella”).

**S-Locus** (“Spotting”); see Table S4f: The *MITF* gene (microphthalmia associated transcription factor) is associated with one or more spotting patterns in dogs [47,55,85]. The range of spotting patterns is large, starting from no spotting and minimal white spotting, known as wild type (S/S), pseudo irish spotting (S/S^p^), to piebald spotting and extremely white spotting (S^p^/S^p^). This is mainly caused by a SINE insertion on the *MITF* gene [85].

**M-Locus** (“Merle”); see Table S4g: The premelanosome protein (*PMEL*) gene is responsible for merle coats described by the following features: (1) a light, diluted base colour and (2) random patches of fully pigmented fur of various size and location [39,40,41]. As the variant is semi-dominant, heterozygous individuals have at least a mild dilution of eumelanin areas, whereas m/m animals are normally pigmented and M/M individuals are mainly white (the latter occasionally exhibit deafness and ocular problems) [79]. A variety of merle phenotypes is known among dog breeds, such the classic, double, hidden, harlequin, cryptic and dilute forms. The hidden form possesses the M allele but the phenotypic merle pattern is invisible due to the epistatic impact of the e allele at the *MC1R* gene, when homozygous [39,41,86].

**H-Locus** (“Harlequin”); Table S4g: The *PSMB7* (Proteasome 20S Subunit β7) gene is a dominant modifier gene of the merle *SILV* gene (M-Locus) that removes the dilute pigment and increases the size of the fully pigmented regions (dark spots on a white background) [37]. Harlequin is only expressed on a Merle background and is a well-known and easily recognizable coat pattern almost exclusively found in Great Danes.

Applying the just-described genetic foundations of coat colour traits to the results of the marker test, in 12 out of 16 analysed dogs, the genotypes predicted the expected phenotypes correctly (Tables S3b and S5).

For six of the 16 dogs, the phenotypic interpretation of the genotypic data obtained was straightforward, and for the other six dogs, heterozygous alleles in Q331 (b^s^) were detected. Three of the latter group had a brown coat colour, one had a black coat colour and one showed a grey coat that is in accordance with the recessive inheritance of the coat colour modifier gene *MLPH*. One dog had an “e/e” *MC1R* genotype and a “B/b^s^” *TYPR1* genotype and showed brown eye rims and brown nose leather, which is in accordance with the description given in [55].

For three dog samples, the sequence analyses failed in the ASIP SINE markers. However, due to the hierarchical gene expression of the coat colour genes, it was deducible that the *ASIP* gene was of no account in these cases. Dog DE02_20 showed an e/e genotype at the E-locus and both dogs DE01_006 and DE01_052 had a K^B^/K^B^ genotype at the K-locus (Figure S2, Table S3b).

The only discrepancy within the group of 16 dogs refers to the genotype interpretation of markers TYRP1_Q331ter and TYRP1_345delP, which refer to a black coat colour instead of actually brown (dog individuals AT0282, DE01_006, DE01_052, and DE02_017).

For the coat pattern trait, consistent results for all tested samples between the genotypic data and the physical visible traits of the dogs were achieved. Only individual AT0134 showed no results in both MITF markers. (Tables S3b and S5).

It should be noted that three dogs (AT0062, AT0183, AT0257) were heterozygous at the K-Locus (K^B^/k^y^), indicating that their coat pattern could be brindled [80], but this pattern was not observed in these individuals. AT0062 showed a black with white spotting phenotype and AT0183 and AT0257 had a solid black phenotypic appearance.

The markers of the remaining trait categories were tested with 10 dog samples each. For each category, the dogs were selected by their appearance, in order to include both manifestations of the particular phenotypic characteristics in question, e.g., smooth vs. curly dogs, small vs. tall dogs.

#### 3.2.2. Coat Structure

Three genes, *RSPO2*, *FGF5*, and *KRT71* (encoding for R-spondin–2, fibroblast growth factor–5, and keratin-71, respectively), together account for most coat structure phenotypes (Table S4h): The *RSPO2* gene is strongly associated with wired hair as well as the “furnishings”, phenotypically characterized by a moustache and pronounced eyebrows as well as increased hair growth on the face and legs [36,87]. Variations in the *FGF5* gene control the hair length in many dog breeds, the short hair mutation being of dominant inheritance [40]. The *KRT71* gene is found to be associated with a curly hair phenotype. As the mutation is semi-dominant, animals heterozygous for this gene have an at least so-called “wavy” coat [40].

The analyses for the three above-mentioned **coat structure trait** markers were straight forward and the genotype data were in accordance with the phenotypical appearance of all 10 dogs (Tables S3b and S5).

#### 3.2.3. Tail Length

The C189G mutation in exon 1 of the *T-Box* transcription factor T gene was found to be strongly correlated with a short-tail phenotype for dogs belonging to specific breeds if heterozygous for this mutation. The T gene mutation can cause both anury (absence of tail) and brachyury (very short tail) [48,54]. Homozygosity for the mutation causes either embryonic or early postnatal lethality [48,88] (Table S4i).

For the prediction of the tail length, the success-rate amounted to 70%. Three of 10 dogs showed no correct genotype–phenotype correlation for the bobtail phenotype (Tables S3b and S5). These samples came from an English Bulldog (AT0056), a Boston Terrier (AT0062) and a French Bulldog (AT0210).

**Table 1:** Detailed information on the selected 21 phenotype-specific markers allocated to the respective trait category. For the coat colour and coat pattern markers, the commonly used locus (Locus) and allele names (Allele) are listed (see, for example, reviews [38,40]). Upper case letters of an allele refer to dominant inheritance, lower case letters to recessive inheritance. For a detailed description of external visible traits (phenotype), see Section 3.2.1, Section 3.2.2 and Section 3.2.3. Additionally, the phenotypic manifestation is given as well as the corresponding references. Sequence information for the marker types INDEL and SINE are given in Table S2b. n.a. not applicable.

Trait Category	Marker ID	Locus	Allele	Marker Type	Nucleotide	Dominance (x)	Phenotype	Reference
Coat colour, coat pattern	MC1R_306ter	E (extension)	e	SNP	T		red, yellow, cream, white	[33,34,35,36,37,38,39,40,41,42,43,44,45,46,47,48,49,50,51,52,53,54,55]
			E		C	x	black, brown	
	**MC1R_M264V**	**E (extension)**	**E**	**SNP**	**A**		**no melanistic mask (black, brown)**	[32,34]
			**E^m^**		**G**	**x**	**melanistic mask (black, brown)**	
	CBD103_S54	K (from ‘dominant black’)	k^y^	INDEL	GGG		yellow—expression of agouti alleles	[28,80]
			K^B^		DelGGG	x	black, brown, blue	
	**CBD103_S53**	**K (from ‘dominant black’)**	**k^y^**	**SNP**	**G**		**yellow—expression of agouti alleles**	[28,80]
			**K^B^**		**C**	**x**	**black, brown, blue**	
	ASIP S82	A (agouti)	A^y^	SNP	T	x	fawn/sable	[27]
			a^w^		G		wild type/agouti (black, brown)	
	**ASIP H83**	**A (agouti)**	**A^y^**	**SNP**	**A**	**x**	**fawn/sable**	[27]
			**a^w^**		**G**		**wild type/agouti (black, brown)**	
	ASIP_SINE	A (agouti)	a^t^	SINE	SINE		tan points (black, brown), tricolour	[29]
			a^w^		no SINE		wild type/agouti (black, brown)	
	**ASIP_R96**	**A (agouti)**	**a^t^**	**SNP**	**C**		**tan points (black, brown), tricolour**	[27]
			**a**		**T**		**recessive black**	
	TYRP1_Q331ter	B (brown)	B	SNP	C	x	black	[33,55]
			b^s^		T		brown	
	**TYRP1_345delP**	**B (brown)**	**B**	**INDEL**	**CCT**	**x**	**black**	[33,55]
			**b^d^**		**DelCCT**		**brown**	
	MLPH_157471_c. -22G>A	D (dilutes eumelanin)	D	SNP	G	x	not diluted pigmentation	[30,56,84]
			d		A		diluted pigmentation	
	**MITF_SNP**	**S (spotting)**	**S**	**SNP**	**A**	**not clarified**	**solid colorred, minimal white spotting**	[47]
			**S**		**G**		**white spotting**	
	MITF_INS	S (spotting)	S	SINE	no SINE		solid coloured, minimal white spotting	[85]
			S^p^		SINE		white spotting (S^p^—piebald)	
			S/S^p^				pseudo irish spotting	
	**PMEL**	**M (merle)**	**M**	**SINE**	**SINE**	**semi dominant**	**merle**	[39,40,41,46]
			**m**		**no SINE**		**no merle**	
			M/m		SINE/no SINE		mild merle	
	PSMB7	H (harlequin)	H	SNP	G	x	harlequin	[35,37]
			h		T		no harlequin	
Coatcoat structure	**FGF5**	**n.a.**	**n.a.**	**SNP**	**G**	**x**	**short hair**	[36]
					**T**		**long hair**	
	RSPO2	n.a.	n.a.	INDEL	no Ins		no furnishings	[36]
					Ins	x	furnishings	
	**KRT71**	**n.a.**	**n.a.**	**SNP**	**A**	**semi dominant**	**smooth coat**	[36,40]
					**G**		**curly coat**	
					**A/G**		**wavy coat**	
Tail length	T-Box_C295G	n.a.	n.a.	SNP	G	x	bobtail	[48,54]
					C		long tail	
Ear shape	**BICFPJ1062878**	**n.a.**	**n.a.**	**SNP**	**G**	**not clarified**	**non drop ears**	[89]
					**A**		**drop ears**	
Body size	IGF1R	n.a.	n.a.	SNP	G		rather tall	[49]
					A	x	rather small	

#### 3.2.4. Ear Shape

Wild canines have upright ears, whereas hanging ears are a characteristic of dog domestication. The marker IGF2BP2_BICFPJ1062878 used for ear shape determination was described in [89] (Table S4j).

The interpretation of this single ear shape marker included in the marker set proved to be unreliable (Tables S3b and S5). Only two dogs reflected the genotype in their phenotypical appearance. One dog did not show drop ears as genotypically indicated. No clear interpretation was possible for the six heterozygote dogs, as three of them had drop ears, and the other three exhibited non-drop ears. For one sample, no sequence data were obtained (AT0274).

#### 3.2.5. Body Size

The insulin-like growth factor 1 receptor (*IGF1R*) has a strong effect on the size of a dog. The allele “A” of the nonsynonymous SNP at 41,849,479 (CanFam 3.1) is a causal mutation for small body size in dogs (Table S4k) and is present in many tiny breeds [49].

*IGF1R* could be successfully genotyped for all dogs in the marker study. Even though only this single body size marker was included in the marker set, an accurate prediction of the body size in seven out of 10 dogs was possible (Tables S3b and S5).

### 3.3. Blind Test

**Figure 2:**
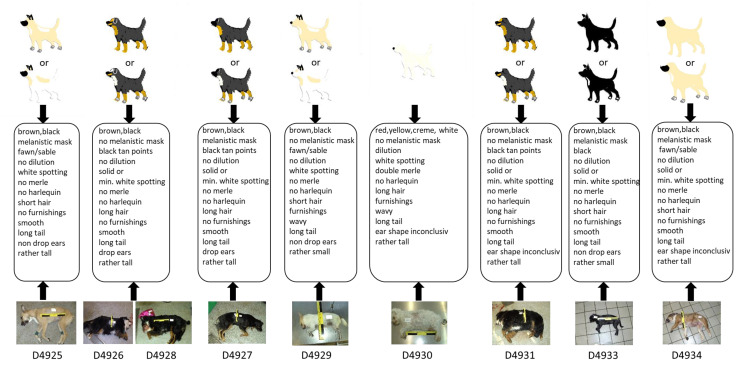
Results of the blind test: Identikit pictures (sketches) were drawn based on the genotypes and subsequently compared with photos of the nine tested dogs. Uncertainties in the manifestation of some visible characteristics required up to two versions of the identikit pictures. For all but one dog (D4930), two versions were necessary in order to reflect the possible phenotypes obtained from the genotype data. Dogs D4926 and D4928 showed the same genotype and therefore identical sketches. In general, identikit pictures and photos showed high levels of similarity. min. = minimal.

In the course of the blind test, nine samples from dogs of blinded phenotypes were analysed with the 21-marker set, resulting in 189 genotypes. For this test, nine canine DNA extracts (D4925–D4931, D4933–D4934) were collected and prepared in a second lab—which also recorded the associated metadata including a photographic documentation—and were sent to the laboratory performing the tests. All samples were successfully genotyped in all markers (Table S3c). Subsequently, sketches of the nine dogs were drawn based on the genetic information obtained and compared to the dogs’ phenotypes as displayed on photos, which were disclosed after the completion of the genetic analyses and the drawing of the sketches. Figure 2 shows a comparison between the photographical documentation and the images drawn based solely on the genetic data. Additionally, the outcome of the test is summarized as a heatmap (Table S5).

Three dogs had an identical genotype for all markers (D4926, D4928 and D4931) except for the ear shape marker, which showed a heterozygous state in D4931. Indeed, their phenotypical appearance was very similar, as all three individuals had black and tan points and minimal white spotting (S/S and a^t^/a^t^) fur, long smooth hair, a long tail, and a rather tall body size. In all three cases, the ear shape could not be traced by the provided photo documentation.

Dog D4927 showed a very similar genotype as the three dogs described above except the two E-locus markers, both being heterozygous, resulting in a correctly predicted “black tan points” fur including a melanistic mask (Figure 2).

For dog D4925, a high genotype–phenotype concordance was observed, except for the tail length. The genotype indicates a long tail, which is in contrast to the tail on the reference photo. However, the tail seemed to be artificially cropped.

For the light-coloured dog, D4929, with obvious upright ears, a high correspondence between genotypic prediction and phenotypic appearance could be achieved.

D4930 showed, in all but two markers, a high concordance between the genotypic predication and phenotypic appearance. The state of the body size marker suggested a tall body size that obviously did not match the dog’s appearance. The heterozygous state at the ear shape marker led to inconclusive phenotypic interpretation.

Five of the six physical visible traits of D4933 were successfully predicted by the genetic data. Only the ear shape—non-drop ears—could not be unambiguously identified based on the reference photo. The K-Locus showed a heterozygous allelic state (K^B^/k^y^), which in some cases would indicate a brindle coat pattern, but the coat pattern of this individual was black with white spotting.

D4934 had a high correspondence between genotypic predication and phenotypic appearance for coat colour, coat pattern and coat structure, but for the body size, tail length, and ear shape, the assignments were discrepant or inconclusive, respectively.

## 4. Discussion

The canine DNA phenotyping approach introduced here builds on established knowledge mainly gained in genome-wide association studies [49,50,90,91,92,93,94] and further advances in canine genetics, in particular, the sequencing of the dog genome [24,95,96,97]. Hence, there was no requirement to search for new candidate markers possibly associated with morphological traits; rather, a panel of published markers was selected, as summarized in Table 1. Genotyping of various visible traits in dogs has already been established in the context of medical research [98] or to support breeding efforts within particular breeds [82,99]. Existing applications of genomic prediction, some of which are commercially available, are mainly intended to verify pedigrees or breeds, to select specific phenotypes according to market demands or studbook policies, and to avoid associated inherited diseases. Obviously, the objectives of these approaches differ from forensic needs. One fundamental issue relates to sample quality. DNA evidence from crime scenes can be notoriously difficult to assess due to the low quantity and/or quality of DNA and the possibility of contamination with other DNA sources or substances (e.g., PCR inhibitors). Therefore, the aims of the pilot test included the optimization of the laboratory workflow and the verification of the published SNP or INDEL positions. This optimization step comprised the redesign of several published PCR primers, resulting in shortened amplicons (Table S2a), which are known to be more successful when analysing degraded DNA [100,101,102]. The successful completion of the pilot test was the methodological basis for the subsequent experiments.

Based on the idea of a proof of concept study, a relatively small selection of 21 genetic markers described in the literature as causative or highly correlated to externally visible traits was used. The reliability of predicting the phenotype was determined in the marker test. Given the relatively small number of markers addressed herein, the ability to differentiate visible traits turned out to be promising. However, the performance of the individual markers to correctly predict particular trait categories varied considerably. The choice of 15 markers selected for the categories coat colour and coat structure proved to be adequate, as shown by the high predictive accuracy for most of the dogs in this study. The coat colour and structure of nearly all dogs was predicted precisely as shown in Table S5. However, the results of the test also suggest that a targeted expansion of the number of markers could improve the predictions. An example for this assertion can be given for the *TYRP1* gene. Schmutz et al. [55] described the three *TYRP1* alleles—Q331ter, 345delP and S41C—that express a brown coat colour when a combination of any two of the recessive *TYRP1* alleles occurs. Our marker set included only the two markers TYRP1_Q331ter and TYRP1_345delP, which might explain the low performance of colour prediction under some allele combinations (Table S3). Therefore, the inclusion of at least a third *TYRP1* marker (allele “b^c^”), a base substitution in exon 2 that causes a serine to cysteine exchange (S41C; c.121T>A), would probably increase the accuracy of coat colour prediction. Furthermore, it is possible that there are additional rare alleles of TYRP1 causing brown that were not detected in our original survey of dogs, but such alleles would likely be quite rare [55]. On the other hand, the category coat structure seems to be nearly perfectly covered by the three markers included in our marker set. In this category, all samples of the marker test and the blind test were correctly assigned. This is promising, as the category coat structure comprised eye-catching traits such as long hairs and curled coats, which were predicted accurately by only three markers.

For testing body size, only the IGF1R marker was applied. The body size from seven out of 10 dogs was predicted correctly based on simple classification “rather small” versus “rather tall”. Hoopes et al. [49] hypothesized that the IGF1R nonsynonymous SNP is a causal mutation for tiny size in dogs. In their study, Allele A was present in many, but not all, of the tiny breeds. This indicates that the inclusion of more body size-associated markers may help to prevent a possible breed-specific bias. Breed-specific genotypes might also be the reason for false predicted tail length in three of 10 tested dogs. Some breeds including English bulldogs and Boston Terriers have bobtails without the mutation at the *T-Box* gene [48]. This could explain the observed discrepancy in samples AT0056 and AT0062, which belong to both breeds, respectively.

The marker with the lowest explanatory power proved to be IGF2BP2_BICFPJ1062878 which was selected to differentiate between drop ears and non-drop ears. Particularly, the heterozygous state, which was established in 60% of the tested dogs, did not allow for firm conclusions.

In a final step, the practical forensic applicability of our approach was evaluated in the blind test, comprising samples from nine dogs. To illustrate the global outcome of the 21-marker set, a sketch of each dog was prepared on bases of the genotypes observed, which included all characteristics deduced from the genetic data. The real phenotypical appearance was disclosed after the completion of the sketches by providing reference photos. A direct comparison between the sketches and the photos showed a high recognition effect, as laid out in Figure 2. The results of the blind test reflected those obtained in the marker test with a very high accuracy for coat colour, coat pattern and coat structure prediction. The discrepancies observed in the remaining traits may be related to the small number of genetic markers used for these traits, particularly for ear shape, body size and tail length. Additionally, the effect of (cosmetic) manipulation was observed as evident in the incorrect tail length prediction (long tail) of dog D4925, which was caused by cropping. Although prohibited in some countries, tail docking cannot be excluded. The same applies to ear cropping, which is also commonly observed. Such artificially altered body parts cannot be predicted but should be taken into consideration when translating the genetic information into a phenotypic sketch.

## 5. Conclusions

This proof of concept study for the first time presents an experimental approach to predict externally visible traits in dogs from DNA for forensic purposes. We used six trait categories that were selected on the bases of an untrained person´s ability to recognize them, as an eyewitness would probably be. The experiments were successful for the majority of the selected markers and predicted traits allowed for a realistic impression of the general appearance of the tested dogs. These promising results stimulate future research that requires an increased set of markers and individuals. We note that an extensive phenotypical documentation of the studied individuals is crucial for the interpretation of the predicted traits. Finally, we suggest the development of molecular genetic tools that allow for a stream-lined laboratory workflow compatible with forensic quality sample types.

## Supplementary Materials

Supporting materials are available online at https://www.mdpi.com/article/10.3390/genes12060908/s1, Figure S1a–d: sanger sequencing chromatograms of dog samples with heterozygous genotypes in length variant markers. The base circled indicates from where it is possible to distinguish alleles with (+) and without (−) the insertion. The arrow indicates the interpretation reading direction. The sequences of all SINEs and the insertion are provided in Table S2b. Table S1a: ASIP SINE, heterozygous state: SINE (−) and SINE (+), Table S1b: MITF SINE, heterozygous state: heterozygote SINE (−) and SINE (+), Table S1c: RSPO2 INS, heterozygous state: Insertion (−) and Insertion (+), Table S1d: PMEL SINE, heterozygous state: SINE (−) and SINE (+). Figure S2: Simplified chart of coat colour loci and alleles in the dog (modified according to Embark Veterinary https://embarkvet.com/breeders/resources/canine-genetics-for-dog-breeders/coat-color/genetics-101/) (accessed on 10 June 2021). It summarizes the dominance hierarchy of loci and alleles involved in the manifestation of coat colour and pattern. The colouring of the boxes (“Loci”) and the coloured circles below the allele designations indicate the deducible coat colours. For simplicity, coat colour modifiers “S-locus” (white spotting), “D-locus” (dilution), “M-locus” (merle), and “H-locus” (harlequin) were not included.

## Appendix A

Collaborators (in alphabetic order) of CaDNAP (Canine DNA Profiling Group): Andreas Hellmann (Bundeskriminalamt, Kriminaltechnisches Institut, Wiesbaden, Germany), Nadja Morf (Institute of Forensic Medicine, University of Zurich, Switzerland), Udo Rohleder (Bundeskriminalamt, Kriminaltechnisches Institut, Wiesbaden, Germany); Uwe Schleenbecker (Bundeskriminalamt, Kriminaltechnisches Institut, Wiesbaden, Germany).
