# Supplementary material for: Towards Forensic DNA Phenotyping for Predicting Visible Traits in Dogs
Source: Genes (Basel). 2021 Jun 11;12(6):908. doi: 10.3390/genes12060908 (PMC8230911; doi:10.3390/genes12060908)
Supplement: Supplementary file 1 [file genes-12-00908-s001.zip › Figure S2.pdf]

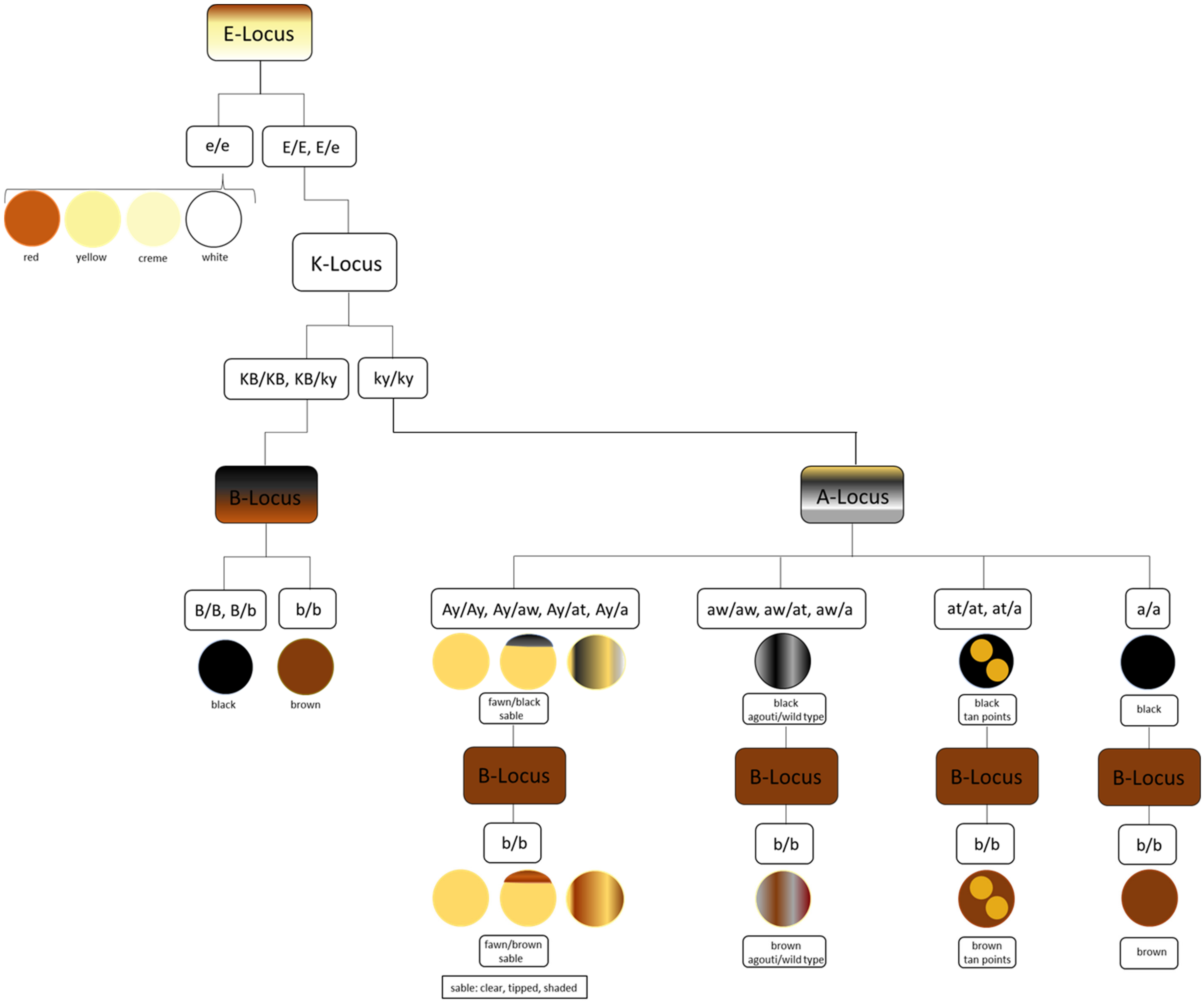

E-Locus
e/e
E/E, E/e
red
yellow
creme
white
K-Locus
KB/KB, KB/ky
ky/ky
B-Locus
A-Locus
B/B, B/b
b/b
black
brown
Ay/Ay, Ay/aw, Ay/at, Ay/a
aw/aw, aw/at, aw/a
at/at, at/a
a/a
fawn/black sable
black agouti/wild type
black tan points
black
B-Locus
B-Locus
B-Locus
B-Locus
b/b
b/b
b/b
b/b
fawn/brown sable
brown agouti/wild type
brown tan points
brown
sable: clear, tipped, shaded

Figure S2: Simplified chart of coat colour loci and alleles in the dog (modified according to Embark Veterinary https://embarkvet.com/breeders/resources/canine-genetics-for-dog-breeders/coat-color/genetics-101/). It summarizes the dominance hierarchy of loci and alleles involved in the manifestation of coat colour and pattern. The colouring of the boxes (“Loci”) and the coloured circles below the allele designations indicate the deducible coat colours. For simplicity coat colour modifiers “S-locus” (white spotting), “D-locus” (dilution), “M-locus” (merle), and “H-locus” (harlequin) were not included.
