# Supplementary material for: Towards Forensic DNA Phenotyping for Predicting Visible Traits in Dogs
Source: Genes (Basel). 2021 Jun 11;12(6):908. doi: 10.3390/genes12060908 (PMC8230911; doi:10.3390/genes12060908)
Supplement: Supplementary file 1 [file genes-12-00908-s001.zip › Figures S1a-S1d .pdf]

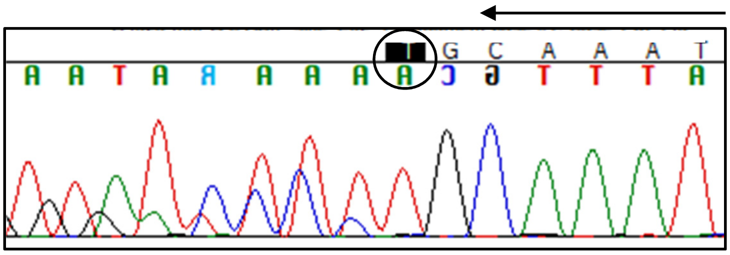

a)

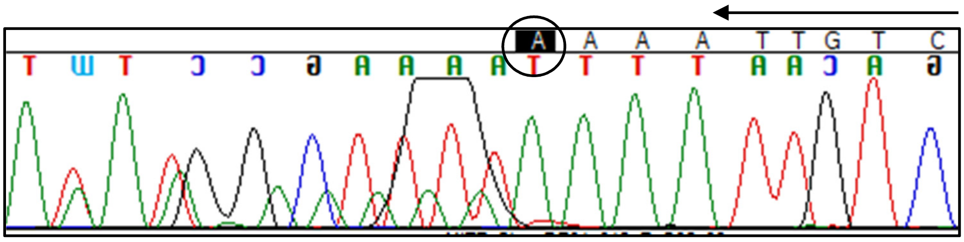

b)

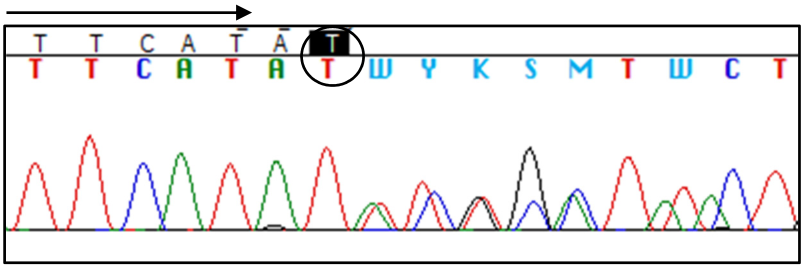

c)

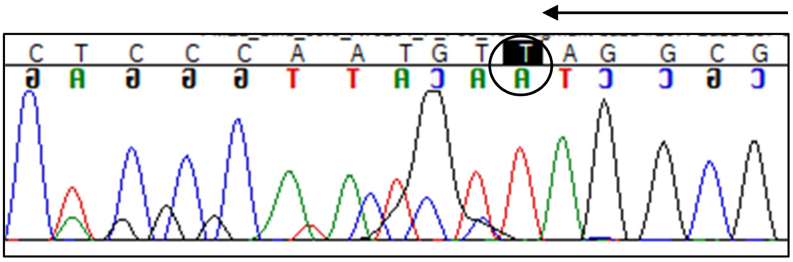

d)

Figure S1a-1d: Sanger Sequencing chromatograms of dog samples with heterozygous genotypes in length variant markers. The base circled indicates from where it is possible to distinguish alleles with (+) and without (-) the insertion. The arrow indicates the interpretation reading direction. The sequences of all SINEs and the insertion are provided in TableS2b.

S1a: ASIP SINE, heterozygous state: SINE (-) and SINE (+)

S1b: MITF SINE, heterozygous state: heterozygote SINE (-) and SINE (+).

S1c: RSPO2 INS, heterozygous state: Insertion (-) and Insertion (+).

S1d: PMEL SINE, heterozygous state: SINE (-) and SINE (+).
